# Vitamin supplement consumption and breast cancer risk: a review

**DOI:** 10.3332/ecancer.2013.365

**Published:** 2013-10-23

**Authors:** Alessandro M Misotti, Patrizia Gnagnarella

**Affiliations:** Division of Epidemiology and Biostatistics, European Institute of Oncology, Milan 20141, Italy

**Keywords:** breast cancer, dietary supplements, vitamin supplementation, vitamins, antioxidants

## Abstract

**Background:**

Breast cancer is the most frequently diagnosed cancer globally, and studies provide contradictory results about the possible effects of vitamin supplementation to reduce cancer risk. Our aim was to conduct a review to better investigate whether vitamin supplements given orally modify breast cancer risk.

**Methods:**

We conducted a comprehensive, systematic bibliographic search of the medical literature to identify relevant studies. Case-control, cohort studies, and randomised controlled trials (RCTs) published up to August 2013 that reported cancer risk estimates for vitamin supplementation were included. For each study, we retrieved study characteristics, study population, exposure evaluation, and risk estimates.

**Results:**

We identified 26 studies (14 cohort, 11 case-control, and one RCT) and overall, we found 104 estimates. We grouped all the estimates into six supplementation categories: vitamin A and beta-carotene, B-group vitamins and folic acid, vitamin C, vitamin D, vitamin E, and multivitamins. Only a few studies showed a statistically significant association between the consumption of supplemental vitamins and the occurrence of breast cancer, and most of the significant estimates were found in case-control studies. The results found in prospective studies seem to be in the opposite direction.

**Conclusion:**

The role of vitamin supplements in preventing breast cancer still remains unclear, considering our review. Although biologic mechanisms exist to support the anticancer effects of vitamins, there is no clear evidence for an effect in cancer prevention for vitamin supplements. Further investigations are warranted to elucidate the mechanisms by which vitamin supplementation can modify breast cancer development.

## Introduction

Breast cancer is the most frequently diagnosed cancer globally and also the main cause of death among women [1]. The highest incidence rates are reported in Western and Northern Europe, Australia, and North America, which is mainly owing to changes in reproductive and hormonal factors, and the availability of screening programs [1]. Breast cancer is a complex disorder and can occur as a result of a process that includes oxidative stress and lipid peroxidation mechanisms [2] beyond gene--environment interactions and hormonal expression [3]. Biologic mechanisms exist to support the anticancer effects of dietary antioxidant vitamins and the overall impression is that intake of some vitamins may play a substantial role in the prevention of cancer [4–6], but the findings are not strong in this regard.

Observational studies and randomised controlled trials provide contradictory results about the possible preventive effects of vitamin supplementation to reduce cancer risk [7–10]. Bjelakovic *et al *[11], in a recent meta-analysis, found no evidence that antioxidant supplements can prevent gastrointestinal cancers; in contrast, they found a possible increasing effect on mortality for excessive consumption. The second World Cancer Research Fund/American Institute for Cancer Research expert report, published in 2007 [6], states that high-dose nutrient supplements can be protective or can cause cancer. The strongest evidence refers to beta-carotene supplementation and lung cancer; actually, high doses of beta-carotene can cause lung cancer in tobacco smokers. The evidence is limited for other vitamins, and there are no recommendations for breast cancer [6].

The use of dietary supplements, such as vitamins and multivitamins, is constantly increasing worldwide [12, 13]. Half of the US adult population (49%) regularly consumes one or more supplements. The use of supplements is different between genders: women tend to report a higher consumption than men (53% versus 44%) [14]. In Canada, the use of supplements is 48% in women and 32% in men [15], while in Australia, habitual consumers of supplements are 27% of women and 15% of men [16]. Women reporting a regular and high consumption of supplements are 41% and 64% in Sweden and in Denmark, respectively [17]. Consumption of dietary supplements of 28% (age: 35–50) and 34% are reported in Germany and Netherlands, respectively [18, 19]. These percentages tend to decrease in central European countries: 24% of the population uses supplements in the United Kingdom (mostly women), and 20% in France [20, 21]. Mediterranean countries present the lowest consumptions: 5% of the population in Italy [22], 0.5% in men and 6.7% in women in Greece [17]; 6.6% in men and 13.4% in women in Spain [17].

In view of evaluating the number of published reports on the relation between supplement use and breast cancer risk, we decided to conduct a review to investigate whether vitamin supplements given orally modify breast cancer incidence in humans.

## Methods

We conducted a comprehensive, systematic bibliographic search of the medical literature to identify relevant studies on vitamin supplementation use and breast cancer risk. A literature search was performed up to August 2013 using the following databases: PubMed, ISIWeb of Science (Science Citation Index Expanded), and Embase. To identify all relevant published papers, our search consisted of a combination of the following MeSH terms: breast neoplasms, dietary supplements, vitamin E, vitamin D, vitamin A, ascorbic acid, vitamin B complex, vitamin B12, carotenoids, folic acid. We also searched for other non-indexed citations, using keywords such as ‘breast’ and ‘vitamin$ or ascorbic’ and ‘supplement$’. The related-records feature was used to amplify the search. In addition, reference lists of relevant articles and reviews were reviewed. The search was limited to human studies but no language or time restrictions were applied. We searched for publications with frequencies or estimates of the relative risk (RR), odd ratio (OR), incidence rate ratio (IRR), and hazard ratio (HR) for breast cancer. We decided to consider, for the review, only cohort studies and case-control studies that analysed the correlation between vitamin supplementation and breast cancer risk. Randomised controlled trials (RCTs) were included whenever they reported analysis on baseline vitamin consumptions, but they were excluded if they provided estimates on experimental vitamin supplementation. Reviews and meta-analyses were not considered eligible but were used to retrieve articles.

Studies reporting the biological mechanism of vitamins in cancer, analysis on dietary vitamins, and supplementation were excluded. We excluded two reports [23, 24] because we found more recent publications based on the same study population [25, 26].

We considered only data about vitamin intake coming exclusively from supplementation. Studies that analysed food plus supplementation intake of vitamins were not considered. We also rejected studies regarding other tumour sites than breast cancer.

For each study, we retrieved the following information:

- Study characteristics: first author, publication year, country and name of the cohort (if available), study design, starting year of the study, type of supplement
- Study population: number of cases and controls or cohort size, age range, menopausal status (if available)
- Exposure evaluation: the upper category of daily intake of supplement (Q max versus the lowest category Q1) or duration in years or users versus non-users
- Statistic: risk estimates, RR, OR, IRR, or HR and 95% confidence intervals (CIs).

## Results

We identified 26 eligible studies to investigate the associations of vitamin supplementations with breast cancer risk. Selected studies were 14 cohort [4, 25–37], 11 case-control studies [2, 12, 38–46], and one RCT [47]. The main characteristics of the reviewed studies are presented in Table 1. Most of the studies (n. 19, 73%) were carried in the United States, two in Canada, three in Europe (three cohorts), and two in Asia. The most recent study was a case-control study carriedout in Pakistan from 2009 [46], and the oldest one is from the NHANES I Cohort (1971) [29]. The largest study is the Women’s Health Initiative Observational Study cohort [35] including more than 160 000 subjects, while the smallest is a nested case-control study reported by Wu *et al* (486 subjects) [42]. Freudenheim *et al *[39] and Lin *et al *[37] analysed women in the premenopausal status, the other eight studies [4, 26, 30–32, 35–37] included women in postmenopausal status, and 12 studies [12, 25, 28, 33, 34, 38, 40, 42–46] analysed both categories. In the other studies, no information about menopausal status was reported. We grouped all the estimates into six categories: vitamin A and beta-carotene, B-group vitamins and folic acid, vitamin C, vitamin D, vitamin E, and multivitamins (Table 2).

### Vitamin A and beta-carotene

We identified seven studies to investigate the associations of vitamin A supplementation with breast cancer risk. Only one case-control study reported a statistically significant increased risk. Moorman *et al *[43] found that any use of supplemental vitamin A can increase the risk of breast cancer (OR = 1.86; 95% CI: 1.00–3.48). Examining the other studies, three cohort studies reported a decrease of cancer risk but this effect is not statistically significant [4, 25, 27]. Similar results were found in two case-control studies reported by Longnecker *et al * [40] and Rohan *et al *[38]. On the contrary, a case-control study by Dorjgochoo *et al *[44] found a not significant increased risk (OR = 1.20; 95% CI: 0.80–1.60) for the longest supplemental use.

For beta-carotene supplementation, we found four studies, two cohort [26, 31] and two case-control [2, 43]. The case-control by Pan *et al * [2] reported a significantly inverse association between high intake of beta-carotene and breast cancer risk (OR = 0.11; 95% CI: 0.01–0.88) in premenopausal women, but this effect was not found in postmenopausal women.

### B-group vitamins

We identified six studies to investigate the associations of vitamin B supplementations with breast cancer risk. The authors reported estimates for single vitamin B, for vitamin B complex, and for folic acid (Table 2).

Maruti *et al *[32] found no association in the VITAL cohort [*vitamin B*_2_: RR = 0.87 (95% CI: 0.72–1.06), *vitamin B*_6_: RR = 0.90 (95% CI: 0.73–1.10), *vitamin B*_12_: RR = 0.96 (95% CI: 0.78–1.17)]. In the Shanghai Breast Cancer Study, Dorjgochoo *et al *[44] evaluated the consumption of vitamin B1, B2, and niacin supplementation, but the results did not support a relationship with breast cancer incidence for the use of supplements (user versus non-user, duration, and frequency of supplementation).

Wu *et al *[42] analysed two cohorts consuming a vitamin B-complex. The results were not statistically significant, and the estimates were contrasting (first cohort, 1974: users versus non-users OR=1.06; second cohort, 1989: OR=0.57).

Regarding folic acid supplementation, few data have been published. Data collected from two cohorts [26, 32] and one case-control [39] study did not indicate any effect. Stolzenberg-Solomon *et al *[47] found an increased risk for a folic acid supplementation greater than 400 µg/day (HR=1.19; 95% CI: 1.01–1.41) in their RCT analysis.

### Vitamin C

We found 11 studies, six cohort [4, 25–28, 31], and five case-control studies (one nested case-control study) [2, 38, 39, 43, 44]. Two studies reported a significant increased risk of breast cancer. Cui *et al *[31] reported an increased risk (RR=1.16; 95% CI: 1.04–1.30) for high supplement intake (≥ 711mg) in a cohort of postmenopausal women. Rohan *et al *[38] found an increased risk (OR=1.46; 95% CI: 1.05–2.01) for high consumption (250 mg) in a case-control. The results were not statistically significant in the other studies also considering long-duration supplement use or frequency of consumption [25, 43, 44].

### Vitamin D

The association between breast cancer risk and vitamin D supplementation has been analysed by three cohort studies [29, 30, 37]. The results suggest a decreased risk of breast cancer, although not significant. We found a statistically significant decreased risk in two case-control studies: Shamsi *et al *[46] found a reduced risk for vitamin D supplementation lasting more than three years (OR=0.27; 95% CI: 0.13–0.56) and Rollison *et al *[45] found a protective effect of more than 400 IU/day vitamin D supplementation (OR=0.79; 95% CI: 0.65–0.96).

### Vitamin E

Five cohort [4, 25–27, 31] and four case-control [38, 39, 43, 44] studies analysed the relation between vitamin E supplementation and the risk of breast cancer. None of them reported an effect on breast cancer risk, except Pan *et al *[2] who reported a protective effect of high intake and long duration of vitamin E supplementation in postmenopausal women (OR=0.64; 95% CI: 0.42–0.99; OR=0.75; 95% CI: 0.58–0.97, respectively).

### Multivitamins

The association between breast cancer risk and multivitamin supplementation has been analysed by six cohort [25, 32–36] and six case-control studies (one nested) [2, 12, 42–44]. The results are contradictory. Larsson *et al *[33] found an increased breast cancer risk associated both with high frequency (RR=1.19; 95% CI: 1.02–1.39) and high duration of multivitamin use (RR=1.22; 95% CI: 1.01–1.47) in the Swedish Mammography Cohort study. Pan *et al *[2] found contrasting results in the National Enhanced Cancer Surveillance System (Canada, case-control study). They found a protective effect for a supplementation lasting more than ten years in postmenopausal women (OR=0.74; 95% CI: 0.59–0.92) but not in premenopausal. In the remaining studies, no associations were found.

## Discussion

In the present review, we examined the intake of vitamin supplements and its association with breast cancer risk. Out of the 26 studies, we identified 104 estimates evaluating the effect of vitamin A and beta-carotene, B-group vitamins and folic acid, vitamin C, vitamin D, vitamin E, and multivitamin. We found 14 significant estimates. This review addresses the supposition that the use of multivitamin or single vitamins does not have an effect on breast cancer risk.

A point to note is that most of the significant estimates were found in a large-population case-control study within the National Enhanced Cancer Surveillance System in Canada [2] (Table 2). Pan *et al* found that supplementations of ten years or longer of multiple vitamins, beta-carotene, vitamin C, and vitamin E were associated with statistically significant reductions for breast cancer risk for postmenopausal women. Moreover, they found a reduced cancer risk also for high intake of beta-carotene in premenopausal women and vitamin E in postmenopausal women. It is known that the oxidative stress and lipid peroxidation are linked to the aetiology of breast cancer, and antioxidants may exert their effect, blocking free radicals, inducing apoptosis in cancer cells, and inhibiting cell proliferation [48–49]. However, it seems that other factors might interfere with cancer development, such as menopausal status. This status is physiological but it produces significant modifications in women (body fat distribution, bone and breast density, ovarian and endometrial changes, cardiovascular modifications). It may influence breast cancer development and/or the interaction between antioxidants and the breast tissue, but it is difficult to fully understand the consequences and the potential clinical implications. Another factor that might interfere with cancer development is the receptor status [estrogen receptor (ER) or progesterone receptor (PR)]. These are the most widely studied markers in breast tissue, and their presence has an important clinical relevance. Cui *et al* found, in the Women’s Health Initiative Observational Study on postmenopausal women, an inverse association between breast cancer risk and dietary carotenoids in subject with ER+ and PR+, but not with other breast cancer groups [31]. However, whether hormone receptor-defined breast cancers are etiologically different is not well understood, and it is difficult to identify an effect linked to the vitamin supplementation. We can underline that the results presented by Pan *et al* can be affected by recall bias inherent in case-control design. Cases might bias their responses to questions on diet after cancer diagnosis. In contrast, cohort and prospective studies have an important advantage because diet assessment is made before diagnosis, and it should be unbiased by cancer experience. The results seem to be in the opposite direction when found in prospective studies (Table 2), and they are also limited, making it difficult to draw any conclusions.

Regarding the use of multivitamins, Larsson *et al *[33] highlighted an increase in the risk of developing breast cancer both for high frequency of consumption (19%) and for long duration of multivitamin supplementation (22%) in the Swedish Mammography cohort study. It is not possible to know the composition of the multivitamins and to separate the effect of the single component for the observed associations. Multivitamins are usually a heterogeneous group of products with no standard composition [2, 33]. The composition may depend on the manufacturer, year of production, and batches [2, 12]. Manufacturers frequently change formulations, and detailed information is not reported [50].

For vitamin C supplementations, we found an increased breast cancer risk of 16% and 46%, respectively, in the Women’s Health Initiative Observational Study cohort [31] and in a nested case-control Study (National Breast Screening) [38]. Regarding vitamin A, only Moorman *et al *[43] in their case-control found an increased risk of breast cancer of 86%, considering any use of supplements. These effects may be due to an increment of oxidative damage [51, 52].

We found an increased risk of breast cancer associated with folic acid supplementation. Stolzenberg-Solomon *et al *[47] found that a supplementation of more than 400 µg/day of folic acid is associated with a 19% increase of the risk of developing breast cancer in the PLCO cancer screening trial. A high folate concentration could contribute to epigenetic changes in gene-regulatory mechanisms, which may result in enhanced cancer development and its promotion. However, results suggest that the role of folic acid in breast cancer development may be more complex, and these mechanisms must be considered with caution [47].

Biological as well as epidemiologic data suggest that vitamin D status could affect cancer risk and play a role in cancer prevention. All the studies included about vitamin D supplementation seem to show a decreased risk in breast cancer, though only two estimates were statistically significant. Rollison *et al *[45] and Shamsi *et al *[46] found a decreased risk of 21% and 73% in case-control studies. The link between vitamin D and breast cancer is based on the concept that the vitamin D receptor (VDR) and its ligand 1,25D (the biologically active form of vitamin D) promotes or maintains the differentiated phenotype in normal mammary cells [53]. Nevertheless, other differences in the vitamin D synthesis from sunlight exposure, circulating vitamin D levels, and ethnicity can affect the association [29] and this may explain the null association found in the other studies.

In addition, we found other differences between studies that made it difficult to compare results. Most of the studies collected information about vitamin supplement consumption throughout food frequency questionnaires, estimating average intakes over the preceding year at baseline, or open-ended questions [28, 33]. Some researchers reconstructed the consumption of supplements over long periods of time [2, 32, 43], estimating average intakes over the 5, 10, 20 years preceding (at baseline) or re-administering the questionnaire (cohort study). Their aim is to study the effect of latency time (time from exposure to cancer diagnosis). The specific time intervals after exposure or the relevant exposure time is unknown in diseases with long incubation periods such as cancer. Therefore, it is difficult to identify the relevant exposure period and antioxidants may exert their effect over a long period of time. The lack of evidence underlines the need to develop a more reliable and standardised method to assess supplement consumption.

Another interesting aspect is the dose of vitamin supplementation reported in selected studies (Table 2). The ‘upper level of intake’ and also the measurement unit are very heterogeneous. For instance, vitamin A doses vary from 5,000 to 23,000 IU/day, while vitamin C supplementation varies from 64 to 1,300 mg/day. Vitamin E varies from 4 to 424 mg/day. Vitamin consumption is reported in milligrams/micrograms or International Units. However, few studies provided evidences of a dose--response relationship between dose of supplementation and breast cancer risk [31, 45, 47]. Moreover, in some studies, authors reported only the frequency of consumption, the duration of use, or the user versus non-user data, not enabling us to make a direct comparison between estimates.

We decided to evaluate only vitamin consumption from supplementation, not including dietary intake. It is known that nutritional status and food intake may vary and may influence cancer risk [6], but we would separate these effects, trying to provide a picture of the role of vitamin supplementation in breast cancer risk. The food is constituted of a wide variety of intended and unintended chemicals which may act singly on human metabolism, but more likely act in groups in a synergistic way. Vitamins and bioactive components may interact between them, not acting in isolation [26, 54, 55].

We retrieved all relevant studies through our systematic search. The data extraction from studies have been performed and checked by individual reviewers, in order not to miss any data. Unfortunately, it should also be noted that some limitations exist. First, the number of studies was relatively small, which may limit our evaluation. We extracted fully adjusted estimates, for known confounding factors, however, in some studies the estimate can be modified by other aspects. It is possible that the observed relationships could be partly due to unmeasured or residual confounding such as other medical conditions or other factors (dietary intake, alcohol intake, sun exposure, lifestyle factors, physical activities, hormone receptor status) that could modify the estimates.

## Conclusion

In conclusion, while dietary factors may be crucial in modifying cancer risk, the role of vitamin supplements in preventing breast cancer still remains unclear, considering our review. Although biologic mechanisms exist to support the anticancer effects of vitamins, this review did not address whether supplement use may have an effect on breast cancer risk. Considering the current recommendations and expert report published [6, 55], there is no clear evidence for an effect in cancer prevention for vitamin supplements due to the fact that few studies have generated relevant data and some results have been contradictory. Further investigations are warranted to elucidate the mechanisms by which vitamin supplementation may modify breast cancer development.

## Conflicts of interest

The authors declare that they have no conflicts of interest.

## Figures and Tables

**Table 1.** Characteristics of the studies included in the review on the association between vitamin supplementations and breast cancer risk.

First author, publication year	Country, study name	Study design	Starting year	Age	N. cases	N. controls / cohort size	Menopausal status	Vitamin supplement
Cui Y, 2008 [31]	USA, Women’s Health Initiative Observational Study	Cohort	1993	50–79	2879	84,805	Post	Beta-carotene, C, E
Dorjgochoo T, 2008 [44]	China, Shangai Breast Cancer Study	Case-control	1996	47–51	3454	3474	Pre + post	A, B-complex, C, E, multivitamin
Feigelson HS, 2003 [36]	USA, Cancer Prevention Study II Nutrition Cohort	Cohort	1992	40–87	1303	66,561	Post	Multivitamin
Freudenheim JL, 1996 [39]	USA, Erie and Niagara counties	Case-control	1986	>40	297	311	Pre	C, E, folate
Ishitani K, 2008 [34]	USA, Women’s Health Study	Cohort	1992	>45	1171	37,920	Pre Post Pre + post	Multivitamin
John EM, 1999 [29]	USA, NHANES I	Cohort	1971	25–74	179	4747	N/D	D
Kushi LH, 1996 [4]	USA, Iowa Women’s Health Study	Cohort	1986	55–69	879	33,017	Post	A, C, E
Larsson SC, 2010 [33]	Sweden, Swedish Mammography Cohort	Cohort	1987	49–83	974	35,329	Pre + post	Multivitamin
Lin J, 2007 [37]	USA, Women’s Health Study	Cohort	1993	>45	276 743	10,302 20,166	Pre Post	D
Longnecker MP, 1997 [40]	USA, Maine, Massachusetts, New Hampshire, Wisconsin	Case-control	1988	<75	3543	9406	Pre + post	A
Maruti SS, 2009 [32]	USA, ‘VITAL Cohort’	Cohort	2000	50–76	743	35,023	Post	B complex, folate, multivitamin
Meulepas JM, 2010 [12]	USA, Wisconsin	Case-control	2004	20–69	2968	2982	Pre Post Pre + post	Multivitamin
Moorman PG, 2001 [43]	USA, Carolina Breast Cancer Study	Case-control	1993	20–74	861	790	Pre + post	A, C, E, beta-carotene, multivitamin
Neuhouser ML, 2009 [35]	USA, Women’s Health Initiative	Cohort + RCT	1993	50–79	4400	161,806	Post	Multivitamin
Pan SY, 2011 [2]	Canada, National Enhanced Cancer Surveillance System	Case-control	1994	20–76	2362	2462	Pre Post	Beta-carotene, C, E, multivitamin
Potischman N, 1999 [41]	USA, Atlanta, Seattle, and New Jersey	Case-control	1990	20–44	568	1451	N/D	D
Robien K, 2007 [30]	USA, Iowa Women’s Health Study	Cohort	1986	61 ±4	2440	34,321	Post	D
Rohan TE, 1993 [38]	Canada, National Breast Screening Study	Nested CC	1982	40–59	519	1182	Pre + post	A, C, E
Rollison DE, 2012 [45]	USA, 4-Corners Breast Cancer Study	Case-control	1999	24–79	2318	2521	Pre + post	D
Roswall N, 2010 [26]	Denmark, ‘Diet, Cancer and Health’	Cohort	1993	50–64	1072	26,224	Post	Beta-carotene, C, E, folate
Shamsi U, 2013 [46]	Pakistan, Karachi	Case-control	2009	18–75	257	514	Pre + post	D
Shibata A, 1992 [27]	USA, California	Cohort	1981	73.8 ±7.4	219	11,580 *	N/D	A, C, E
Stolzenberg-Solomon RZ, 2006 [47]	USA, PLCO Cancer Screening Trial	RCT	1993	55–74	691	25,400	Post	Folate, multivitamin
Verhoeven DTH, 1997 [28]	Netherlands	Cohort	1986	55–69	607	1.598	N/D	C
Wu K, 1999 [42]	USA, Washington County	Nested CC	1974	—	133	133	Pre + post	B complex, multivitamin
			1989	—	110	110	Pre + post	
Zhang S, 1999 [25]	USA, Nurses’ Health Study	Cohort	1980	30–55	2523	77,925	Pre + post	A, C, E, multivitamin

* Cohort size includes men and women. N/D: menopausal status not defined. Pre: only women in premenopausal status. Post: only women in postmenopausal status. Pre + post: both women in pre- and postmenopausal status were included in the analysis.

**Table 2.** Risk estimates for the association of breast cancer risk with vitamin supplementation by highest level of intake versus lowest or non-users

First author, publication year	Study design	Exposure: highest level of intake versus non-users	Menopausal status	Risk type	Risk (95% CI)
Vitamin A					
Shibata, 1992 [27]	C	Users	N/D	RR	0.94 (0.72–1.23)
Kushi, 1996 [4]	C	> 10,000 IU/day	Post	RR	0.71 (0.47–1.06)
Zhang, 1999 [25]	C	≥ 23,000 IU/day	Pre + post	RR	0.49 (0.20–1.18)
Zhang, 1999 [25]	C	≥ 10 years supplementation	Pre + post	RR	0.97 (0.59–1.62)
Rohan, 1993 [38]	NCC	> 5000 IU/day	Pre + post	OR	0.70 (0.42–1.15)
Longnecker, 1997 [40]	CC	> 12,000 IU/day	Pre + post	OR	0.40 (0.15–1.05)
Moorman, 2001 [43]	CC	Any use	Pre + post	OR	**1.86 (1.00–3.48)**
Dorjgochoo, 2008 [44]	CC	Longest duration: ≥ 3 years	Pre + post	OR	1.20 (0.80–1.60)
Dorjgochoo, 2008 [44]	CC	Users	Pre + post	OR	1.00 (0.80–1.20)
Dorjgochoo, 2008 [44]	CC	Frequency of consumption: >once daily	Pre + post	OR	0.90 (0.50–1.60)
**Beta-carotene**					
Cui, 2008 [31]	C	≥ 5443 µg/day	Post	RR	1.07 (0.95–1.21)
Roswall, 2010 [26]	C	>8991 µg/day^1^	Post	IRR	1.33 (0.75–2.34)
Moorman, 2001 [43]	CC	Any use	Pre + post	OR	1.37 (0.72–2.60)
Pan, 2011 [2]	CC	≥ 10 years supplementation	Pre	OR	0.51 (0.24–1.06)
Pan, 2011 [2]	CC	≥ 10 years supplementation	Post	OR	**0.58 (0.36–0.95)**
Pan, 2011 [2]	CC	High intake^1^	Pre	OR	**0.11 (0.01–0.88)**
Pan, 2011 [2]	CC	High intake^1^	Post	OR	0.47 (0.20–1.08)
**B-group vitamins**					
Maruti, 2009 (B2) [32]	C	B_2_: 1,70 mg/day (per 10 years)	Post	RR	0.87 (0.72–1.06)
Maruti, 2009 (B6) [32]	C	B_6_: 10,7 mg/day (per 10 years)	Post	RR	0.90 (0.73–1.10)
Maruti, 2009 (B12) [32]	C	B_12_: 25,0 µg/day (per 10 years)	Post	RR	0.96 (0.78–1.17)
Wu, 1999 (cohort 1974) [42]	NCC	Users	Pre + post	OR	1.06 (0.47–2.38)
Wu, 1999 (cohort 1989) [42]	NCC	Users	Pre + post	OR	0.57 (0.27–1.21)
Dorjgochoo, 2008 [44]	CC	≥ 3 years supplementation	Pre + post	OR	1.10 (0.90–1.50)
Dorjgochoo, 2008 [44]	CC	Users	Pre + post	OR	1.00 (0.80–1.20)
Dorjgochoo, 2008 [44]	CC	Frequency of consumption: >once daily	Pre + post	OR	1.00 (0.70–1.40)
**Folic acid**					
Maruti, 2009 [32]	C	400–1400 µg/day	Post	RR	1.00 (0.84–1.19)
Roswall, 2010 [26]	C	>150 mg/day^1^	Post	IRR	0.99 (0.78–1.25)
Freudenheim, 1996 [39]	CC	≥ 400 µg/day	Pre	OR	0.97 (0.67–1.42)
Stolzenberg-Solomon RZ, 2006 [47]	RCT	≥ 400 µg/day	Post	HR	**1.19 (1.01–1.41)**
**Vitamin C**					
Shibata, 1992 [27]	C	Users	N/D	RR	0.93 (0.71–1.23)
Kushi, 1996 [4]	C	> 1.000 mg/day	Post	RR	0.77 (0.50–1.17)
Verhoeven, 1997 [28]	C	Users	N/D	RR	1.06 (0.79–1.43)
Zhang, 1999 [25]	C	≥1.300 mg/day	Pre + post	RR	1.04 (0.77–1.42)
Zhang, 1999 [25]	C	≥10 years supplementation	Pre + post	RR	0.98 (0.83–1.15)
Cui, 2008 [31]	C	≥711 mg	Post	RR	**1.16 (1.04–1.30)**
Roswall, 2010 [26]	C	>64 mg/day^1^	Post	IRR	0.96 (0.77–1.21)
Rohan, 1993 [38]	NCC	> 250 mg/day	Pre + post	OR	**1.46 (1.05–2.01)**
Freudenheim, 1996 [39]	CC	≥264 mg/day	Pre	OR	0.98 (0.62–1.54)
Moorman, 2001 [43]	CC	> 3 years supplementation	Pre + post	OR	1.00 (0.62–1.62)
Moorman, 2001 [43]	CC	Any use	Pre + post	OR	0.88 (0.64–1.19)
Dorjgochoo, 2008 [44]	CC	≥3 years supplementation	Pre + post	OR	0.90 (0.70–1.20)
Dorjgochoo, 2008 [44]	CC	Users	Pre + post	OR	0.90 (0.80–1.10)
Dorjgochoo, 2008 [44]	CC	Frequency of consumption: >once daily	Pre + post	OR	0.90 (0.70–1.20)
Pan, 2011 [2]	CC	≥10 years supplementation	Pre	OR	0.75 (0.54–1.03)
Pan, 2011 [2]	CC	≥ 10 years supplementation	Post	OR	**0.79 (0.63–0.99)**
Pan, 2011 [2]	CC	High intake^1^	Pre	OR	0.70 (0.44–1.10)
Pan, 2011 [2]	CC	High intake^1^	Post	OR	0.85 (0.61–1.17)
**Vitamin D**					
John, 1999 [29]	C	Daily supplement intake	N/D	RR	0.89 (0.60–1.32)
Lin J, 2007 [37]	C	≥400 IU/day	Pre	HR	0.76 (0.50–1.17)
Lin J, 2007 [37]	C	≥400 IU/day	Post	HR	0.87 (0.68–1.12)
Robien, 2007 [30]	C	≥800 IU/day	Post	RR	0.89 (0.74–1.08)
Potischman, 1999 [41]	CC	≥ 400 IU/day	N/D	OR	0.98 (0.80–1.20)
Rollison DE, 2012 [45]	CC	≥ 400 IU/day	Pre + post	OR	**0.79 (0.65–0.96)**
Shamsi U, 2013 [46]	CC	≥ 3 years supplementation	Pre + post	OR	**0.27 (0.13–0.56)**
**Vitamin E**					
Shibata, 1992 [27]	C	Users	N/D	RR	0.89 (0.68–1.16)
Kushi, 1996 [4]	C	> 250 IU/day	Post	RR	0.96 (0.76–1.23)
Zhang, 1999 [25]	C	≥ 600 IU/day	Pre + post	RR	0.92 (0.70–1.21)
Zhang, 1999 [25]	C	≥ 10 years supplementation	Pre + post	RR	1.11 (0.92–1.35)
Cui, 2008 [31]	C	≥ 424 mg	Post	RR	1.01 (0.90–1.14)
Roswall, 2010 [26]	C	>10 mg/day^1^	Post	IRR	1.02 (0.79–1.30)
Rohan, 1993 [38]	NCC	> 4 mg/day	Pre + post	OR	1.00 (0.65–1.54)
Freudenheim, 1996 [39]	CC	α–tocopherol: ≥ 31 mg/day	Pre	OR	0.95 (0.58–1.55)
Moorman, 2001 [43]	CC	> 3 years supplementation	Pre + post	OR	0.78 (0.46–1.35)
Moorman, 2001 [43]	CC	Any use	Pre + post	OR	0.95 (0.68–1.32)
Dorjgochoo, 2008 [44]	CC	≥ 3 years supplementation	Pre + post	OR	0.90 (0.70–1.20)
Dorjgochoo, 2008 [44]	CC	Users	Pre + post	OR	0.90 (0.80–1.10)
Dorjgochoo, 2008 [44]	CC	Frequency of consumption: >once daily	Pre + post	OR	0.90 (0.70–1.20)
Pan, 2011 [2]	CC	≥ 10 years supplementation	Pre	OR	0.74 (0.47–1.17)
Pan, 2011 [2]	CC	≥10 years supplementation	Post	OR	**0.75 (0.58–0.97)**
Pan, 2011 [2]	CC	High intake^1^	Pre	OR	0.72 (0.33–1.59)
Pan, 2011 [2]	CC	High intake^1^	Post	OR	**0.64 (0.42–0.99)**
**Multivitamin**					
Zhang, 1999 [25]	C	≥10 years supplementation	Pre + post	RR	0.96 (0.85–1.09)
Feigelson HS, 2003 [36]	C	Any use in 1982 and 1992	Pre	RR	1.02 (0.89–1.17)
Ishitani K, 2008 [34]	C	≥20 years of supplementation (current users)	Pre + post	RR	1.00 (0.74–1.35)
Ishitani K, 2008 [34]	C	≥6 times/week (current users)	Pre + post	RR	1.00 (0.86–1.16)
Ishitani K, 2008 [34]	C	Current users	Pre + post	RR	0.99 (0.82–1.19)
Ishitani K, 2008 [34]	C	≥20 years of supplementation (current users)	Pre	RR	1.62 (0.81–3.24)
Ishitani K, 2008 [34]	C	≥ 6 times/week (current users)	Pre	RR	1.04 (0.75–1.44)
Ishitani K, 2008 [34]	C	Current users	Pre	RR	1.36 (0.85–2.18)
Ishitani K, 2008 [34]	C	≥20 years of supplementation (current users)	Post	RR	0.77 (0.53–1.13)
Ishitani K, 2008 [34]	C	≥ 6 times/week (current users)	Post	RR	0.91 (0.76–1.10)
Ishitani K, 2008 [34]	C	Current users	Post	RR	0.82 (0.66–1.03)
Maruti, 2009 [32]	C	7 days/week	Post	RR	0.87 (0.72–1.06)
Neuhouser ML, 2009 [35]	C + RCT	Users	Post	HR	1.05 (0.89–1.25)
Neuhouser ML, 2009 [35]	C + RCT	> 10 years supplementation	Post	HR	1.03 (0.94–1.14)
Larsson, 2010 [33]	C	≥7 tablets/week	Pre + post	RR	**1.19 (1.02–1.39)**
Larsson, 2010 [33]	C	≥3 years supplementation	Pre + post	RR	**1.22 (1.01–1.47)**
Wu, 1999 (cohort 1974) [42]	NCC	Users	Pre + post	OR	1.25 (0.67–2.36)
Wu, 1999 (cohort 1989) [42]	NCC	Users	Pre + post	OR	0.77 (0.42–1.43)
Moorman, 2001 [43]	CC	> 3 years supplementation	Pre + post	OR	0.83 (0.59–1.17)
Moorman, 2001 [43]	CC	Any use	Pre + post	OR	0.95 (0.74–1.20)
Dorjgochoo, 2008 [44]	CC	≥3 years supplementation	Pre + post	OR	1.30 (0.90–1.90)
Meulepas JM, 2010 [12]	CC	≥10 years supplementation (current users)	Pre + post	OR	1.13 (0.93–1.38)
Meulepas JM, 2010 [12]	CC	Current users	Pre + post	OR	1.02 (0.87–1.19)
Meulepas JM, 2010 [12]	CC	≥7 times/week	Pre + post	OR	1.00 (0.77–1.28)
Meulepas JM, 2010 [12]	CC	≥10 years of supplementation (current users)	Pre	OR	1.02 (0.78–1.34)
Meulepas JM, 2010 [12]	CC	≥7 times/week	Pre	OR	0.93 (0.66–1.30)
Meulepas JM, 2010 [12]	CC	Current users	Pre	OR	0.87 (0.70–1.08)
Meulepas JM, 2010 [12]	CC	≥10 years of supplementation (current users)	Post	OR	1.13 (0.93–1.38)
Meulepas JM, 2010 [12]	CC	≥ 7 times/week	Post	OR	1.00 (0.77–1.28)
Meulepas JM, 2010 [12]	CC	Current users	Post	OR	1.03 (0.88–1.20)
Pan, 2011 [2]	CC	≥10 years supplementation	Pre	OR	0.93 (0.69–1.27)
Pan, 2011 [2]	CC	≥10 years supplementation	Post	OR	0.74 (0.59–0.92)
Stolzenberg-Solomon RZ, 2006 [47]	RCT	Every use	Post	HR	1.18 (0.95–1.48)

C: cohort study. CC: case-control study. NCC: nested case-control. RCT: randomised controlled trial.*1*: reference group: lowest intake.
